# Functionally Enigmatic Genes: A Case Study of the Brain Ignorome

**DOI:** 10.1371/journal.pone.0088889

**Published:** 2014-02-11

**Authors:** Ashutosh K. Pandey, Lu Lu, Xusheng Wang, Ramin Homayouni, Robert W. Williams

**Affiliations:** 1 UT Center for Integrative and Translational Genomics and Department of Anatomy and Neurobiology, University of Tennessee Health Science Center, Memphis, Tennessee, United States of America; 2 St. Jude Children's Research Hospital, Memphis, Tennessee, United States of America; 3 Department of Biological Sciences, Bioinformatics Program, University of Memphis, Memphis, Tennessee, United States of America; The Centre for Research and Technology, Hellas, Greece

## Abstract

What proportion of genes with intense and selective expression in specific tissues, cells, or systems are still almost completely uncharacterized with respect to biological function? In what ways do these functionally enigmatic genes differ from well-studied genes? To address these two questions, we devised a computational approach that defines so-called *ignoromes*. As proof of principle, we extracted and analyzed a large subset of genes with intense and selective expression in brain. We find that publications associated with this set are highly skewed—the top 5% of genes absorb 70% of the relevant literature. In contrast, approximately 20% of genes have essentially no neuroscience literature. Analysis of the ignorome over the past decade demonstrates that it is stubbornly persistent, and the rapid expansion of the neuroscience literature has not had the expected effect on numbers of these genes. Surprisingly, ignorome genes do not differ from well-studied genes in terms of connectivity in coexpression networks. Nor do they differ with respect to numbers of orthologs, paralogs, or protein domains. The major distinguishing characteristic between these sets of genes is date of discovery, early discovery being associated with greater research momentum—a genomic bandwagon effect. Finally we ask to what extent massive genomic, imaging, and phenotype data sets can be used to provide high-throughput functional annotation for an entire ignorome. In a majority of cases we have been able to extract and add significant information for these neglected genes. In several cases—*ELMOD1, TMEM88B*, and *DZANK1*—we have exploited sequence polymorphisms, large phenome data sets, and reverse genetic methods to evaluate the function of ignorome genes.

## Introduction

The distribution of research on genes is highly skewed and is made up of a small set with dense literature coverage and a larger set with minimal coverage [1]. The distribution follows a power law [2], probably because researchers tend to work collaboratively and collectively on genes they perceive to be important [3]. Pfeiffer and Hoffmann highlight the social bias that can produce this type of bandwagon effect, but they also point to the opposite tendency to explore the unknown. The disappointing fact is that after two decades of intense and sophisticated molecular and genetic analyses, more than a third of all protein-coding genes have almost no literature or known function. We refer to this subset of the genome as an ignorome. If “real knowledge is to know the extent of one's ignorance” (paraphrasing Confucius, analects 2∶17), then we currently need to determine the extent of the ignorome and whether it arises from social patterns of research or unusual biological properties.

An ignorome can be computed at the level of an entire genome, but as we show here, an ignorome can also be computed at the level of a single organ, tissue, or cell type using patterns and levels of expression as a metric of functional importance. These more refined ignoromes are particularly useful when the goal is to define gene or protein function. In the present study we focus on the mammalian CNS—mainly of mouse and human. We have computed ignorome scores that are a function of (1) gene expression in the brain, (2) selectivity of expression in brain versus other organs, and (3) the size of the neuroscience literature. Two steps are essential to define an ignorome using this quantitative approach: accurate assignment of expression data to the correct gene, and precise estimation of the neuroscience literature coverage for each gene. For the first step we used deep RNA-seq data that we recently generated for whole brain [4]–[6] to correctly assign genes in large expression data sets. For the second step, we used a generalizable data mining approach that minimizes irrelevant and false literature linkages created by ambiguous gene names. Using these methods we have been able to extract the most interesting fraction of the ignorome that has both *i*ntense and highly *s*elective *e*xpression (ISE) in the brain. These genes are likely to have greater impact on brain function and behavior, and can be more readily evaluated using genetic, genomic, and phenotypic resources.

Remarkably, we find that the ignorome does not differ from well-studied genes in terms of connectivity in coexpression networks. Numbers of orthologs, paralogs, or protein domains do not differ greatly between the two types of genes. This strongly supports the idea that the neglected subset of the genome is more of a matter of the sociology of science that of inherent differences in biology. Supporting this hypothesis we find that well-studied genes have much earlier discovery dates by an average of 10 years.

We used multiple genomic resources and a variety of approaches to provide a useful functional context for a significant majority of brain ignorome genes including: gene coexpression analysis, protein domain comparison, analysis of in situ expression in many CNS cell types, and a novel genetic method referred to as reverse complex trait analysis [6], [7]. We close by giving several specific and intriguing examples of ignorome genes likely to be of importance in CNS function. The methods we have developed here can be applied widely to prioritize genes for detailed functional analysis in many different tissues, cells, and systems.

## Results

### Genes with intense and selective expression in the brain

To extract the list of genes with intense and selective expression we compared patterns of gene expression in the brain with those in more than 20 other organs and tissue types using both Illumina MouseWG-6 v2 and Affymetrix M430 2.0 arrays. We selected all assays of mRNA expression—probes and probe sets—that had at least eight-fold higher expression in brain compared to other tissues (Methods). We identified 493 and 615 probes and probe sets with intense and selective expression (ISE) in brain, respectively. While we have mostly exploited murine data sets, in the text below, we have used human gene symbols whenever possible.

#### Annotation of intense and selectively expressed genes

Many probes that target specific transcripts in the brain are still poorly annotated, and as a result probes that actually target well-studied genes can be incorrectly nominated as part of an ignorome. We used deep RNA sequencing (RNA-seq) for whole brain (∼1 billion strand-specific tags from 34 strains of mice, see Methods) to reevaluate the annotation for a small subset of ambiguous probes and their associated transcripts (*n* = 28, Table S1). For example, Affymetrix probe set *1457743_at* was not assigned to any gene in the original annotation file, but actually maps to the distal 3′ UTR of *RGS7BP*. Similarly, probe set *1443205_at* was initially assigned to the ignorome gene *D5Buc30e*, but correctly maps to the distal 3′ UTR of *GABRB1* (Figure S1).

We removed redundant probes and retained 406 and 478 ISE genes in the two data sets (Table S2 and S3), and a union of ∼650 genes. Not surprisingly, this set of 650 genes contains intensively studied genes known to be involved in brain function and development. With the exception of *Zfp941*, all 650 have unequivocal human orthologs and also have supporting identifiers in different databases including Refseq, MGI, and Ensemble. More than 98% are conserved across five or more species. A few genes, such as *CMTM5* and *PRR18*, appear to be restricted to mammals. Many others, such as *SGTB* and *RNFT2*, are conserved in more than 10 species (*n* = 170 genes). We have used the union of the two ISE sets as starting material to define a brain ignorome.

### Neuroscience-specific literature coverage

**Figure 1:**
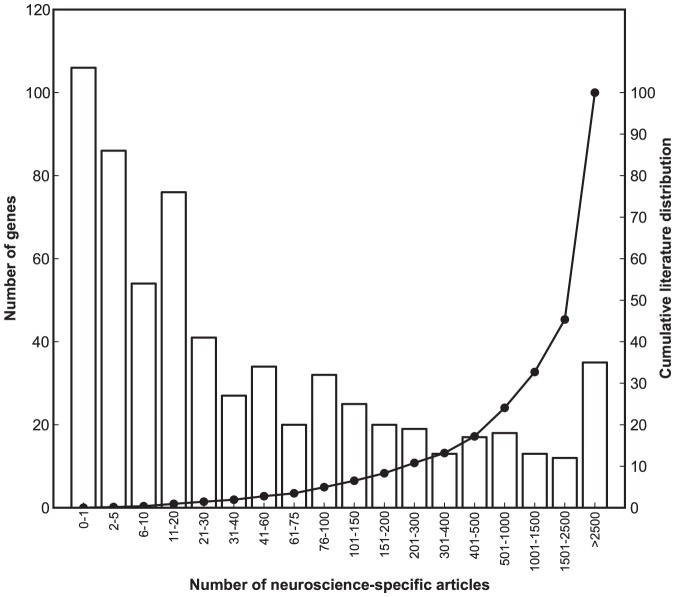
Literature density of 648 genes with intense and selective expression in the brain. The x-axis represents the number of neuroscience-related references in PubMed as of February 2013. The left y-axis represents the number of genes. The right y-axis represents the cumulative literature distribution for ISE genes. A subset of ∼30 genes to the far right (last two bars) absorb two-thirds of the total neuroscience literature. In contrast, ∼200 genes to the far left (first two bars) absorb only 0.1% of the literature.

**Figure 2:**
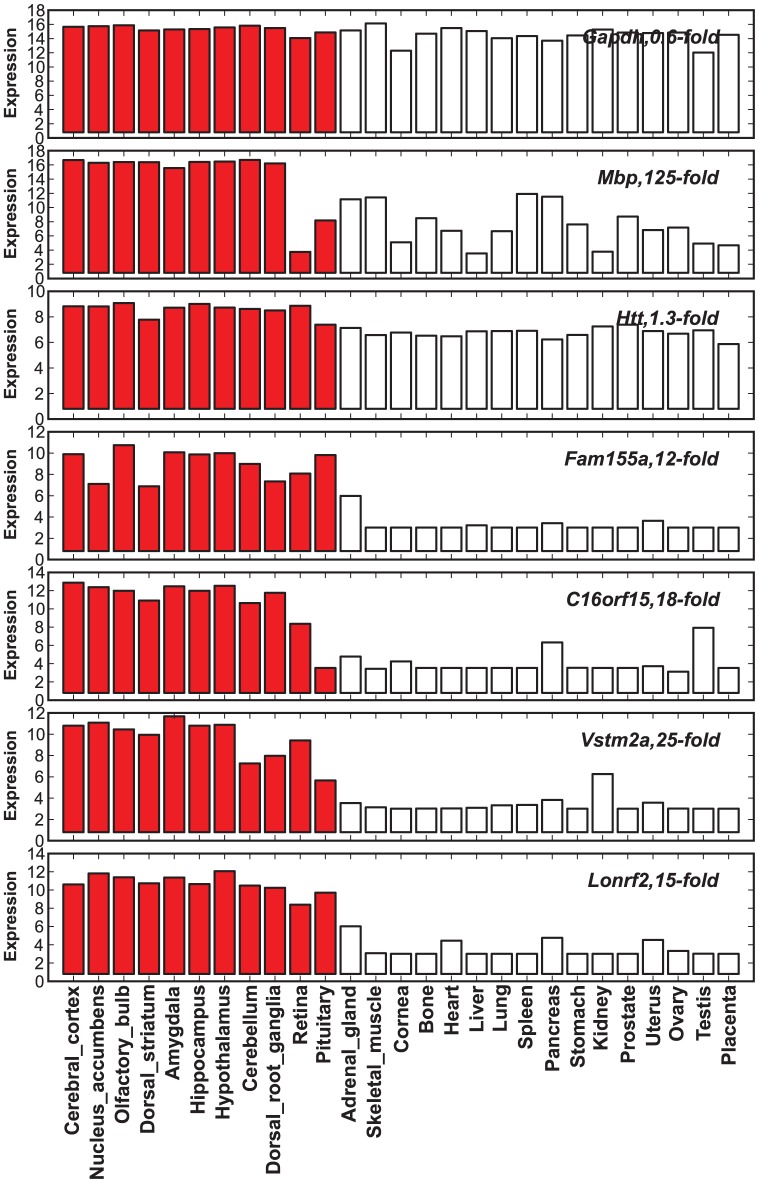
Expression comparison between a few well-studied and ignorome genes in multiple tissues and organs. The x-axis represents multiple tissues and organs including several brain regions represented by red colored bars. y-axis represents the log2 transformed expression values downloaded from BioGPS (www.biogps.org). We included interesting examples including (1) *GAPDH*, a house keeping gene, (2) *MBP*, an extensively studied gene with high brain selectivity, (3) *HTT (HD)*, an important neuroscience gene with low brain selectivity, and ignorome genes including *FAM155A*, *C16orf15*, *VSTM2A*, and *LONRF2* with highly selective expression in brain.

The number of publications for these 650 ISE genes is highly skewed (Figure 1). The top 5% account for ∼68% of the relevant literature whereas the bottom 50% of genes account for only 1% of the literature. Forty-six genes including *GFAP, MBP, VIP, GRIA1*, and *GRIA2* are associated with over 1000 PubMed citations each. In contrast, approximately one-sixth of these genes comprise the core brain ignorome, and are associated with fewer than one paper each (38 papers total). One-tenth of these ISE genes are not associated with any citations and are part of an absolute ignorome. A sample of genes that have no neuroscience publications as of February 2013 include *FAM155A*, *C16orf45*, *C8orf46, VSTM2A, PNMAL1, LONRF2, PGBD5*, and *MAP7D2*. Their high brain expression selectivity (Figure 2) was confirmed independently using multiple sources (Methods), with the expected exception that several have high expression in pituitary, retina, and more rarely, testis.

There are a number of technical issues associated with precisely determining the neuroscience literature coverage of ISE genes. We briefly discuss some steps used to minimize the retrieval of irrelevant literature.

1. *Ambiguity associated with gene names*: Ambiguity of gene names—symbols, aliases and other designations—adds significant error to estimates of literature coverage derived from PubMed [8]. We used a simple disambiguation approach that combines ambiguous gene names with informative and gene-specific key words (Methods). For example, *NSE* is an alias for *ENO2*, a neuronal enolase. However, *NSE* also stands for “normal squamous epithelia” and “neutron spin-echo”. By combining “*NSE*” with “enolase” to produce a joint search, we were able to disambiguate PubMed analysis for this gene.
2. *Neuroscience-restricted search*: The literature for many ISE genes is not restricted to neuroscience. For example, pleiotrophin (*PTN*), a neurotrophic factor for spinal motor neurons, has been studied primarily in the context of bone morphogenesis [9], [10]. As described in Methods, we developed a simple algorithm that enables us to restrict PubMed searches to a specific literature subset—in this case a neuroscience-specific subset.
3. *Inflation due to a noisy or uninformative literature*: Large-scale genomic studies often list hundreds of genes but provide only generic and cursory annotations. Notable examples include studies representing genome-wide surveys such as “The transcriptional landscape of the mammalian genome” [11]. We generated a list of approximately 850 of these articles (Methods) and excluded them from downstream analysis.

### Functional characterization of the ignorome

The ignorome score is a function of both literature coverage information and expression selectivity and ranges from 0 (relatively well known) to 1 (relatively unknown, Table S4).We have defined the core brain ignorome as those genes that have no more than a single neuroscience article in PubMed (Table S4). One-hundred and six out of 650 ISE genes belong to this core ignorome. One-tenth still retain their original names given by the RIKEN Mouse Gene Encyclopedia project [12]. Other subsets belong to the solute carrier family (*SLC39A12, SLC35F3, SLC35F1*), the TAFA family [13] (*FAM123C, FAM131B, FAM155A, FAM171B, FAM189A1, FAM19A1, FAM19A2*, and *FAM81A*), and the family of transmembrane proteins (*TMEM130, TMEM145, TMEM178A, TMEM179, TMEM59L, TMEM88B, TMEM91*).

**Figure 3:**
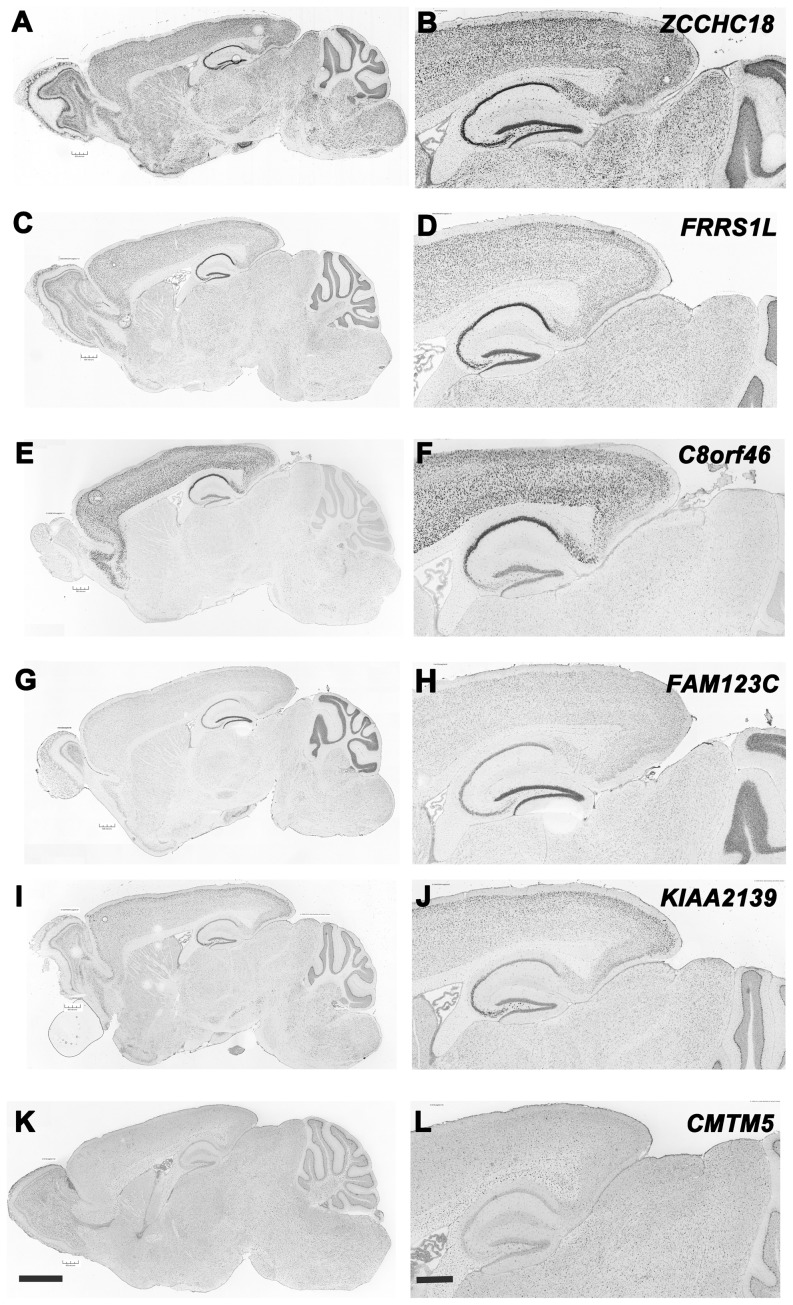
Expression of ignorome transcripts in the C57BL/6J brain. Pairs of low (scale on the lower left represents 1500 microns) and high (scale on the lower right represents 750 microns) resolution images of C57BL/6J brain sagittal sections (labeled using *in situ* probes) for six ignorome gene members taken from the Allen Brain Atlas (http://www.brain-map.org/). A) and B) are low and high resolution images showing *in situ* expression of *ZCCHC18* in sagittal sections. C) and D) show expression of *FRRS1L*. E) and F) show expression of *C8orf46* and G) and H) show expression of *FAM123C*, I) and J) show expression of *KIAA2139*, and K) and L) show expression of *CMTM5*.

Calculation of the ignorome score relied on whole brain gene expression, and as expected 95% of the core has both intense and relatively uniform expression across many brain regions. However, there are interesting exceptions (Figure 3): (1) *KIAA1239* has extraordinarily high and selective expression in Purkinje cells, habenula, pyriform cortex, superficial layer 2 of neocortex, and CA3 of hippocampus; (2) *C8orf46* has extremely high expression only in cortex (archi-, paleo- and neocortex) but low expression in layer 4 of neocortex and part of CA3; (3) *FAM123C* has highest expression in granule cells of cerebellum, dentate gyrus, and olfactory bulbs, and finally, (4) *FRRS1L* has highest expression in Purkinje cells, neocortex, and CA1–3. We also used *in situ* hybridization images downloaded from the Allen Brain Atlas [14] to evaluate patterns of expression in subsets of easily resolvable cell types (e.g., Purkinje cells, Bergmann glia, astrocytes, dentate gyrus granule cells, pyramidal neurons, and white matter oligodendrocytes). Again, more than 95% of ignorome genes have high expression in neurons (e.g., *ZCCHC18* has pan-neuronal expression in Figure 3) and comparatively modest, if any, expression in oligodendrocyte or astrocyte. Only two ignorome genes (*SLC39A12, CMTM5*) are expressed preferentially in astrocytes, Bergmann glia, and oligodendrocytes (Figure 3).

Eighty-five percent of the core ignorome contains known protein domains cataloged in InterPro [15] or Pfam [16]. As shown in Table S5, common domains include leucine-rich repeats (*SLITRK4, LRRC40, LRRC24*), C2 calcium-dependent membrane targeting domains (*SYT16, CPNE9, CPNE4*), small GTPase domains (*DIRAS2, RAB9B, DIRAS1*), and zinc finger motifs (*Zfp941, RNFT2, ZMAT4, LONRF2, ZCCHC18*, and *DZANK1*).

A subset of core ignorome genes (*n* = 10) have known orthologs in worm. With few exceptions, such as *SLITRK4* and *SMAP1* that are associated with embryonic lethality in worms, we were unfortunately not able to add significant functional annotation using this comparative approach and exploiting WormBase [17].

Gene function can often be predicted from patterns of coexpression—guilt by association. We extracted the top 100 covariates of each member of the core ignorome and carried out gene ontology (GO) enrichment analysis using the hypergeometric distribution (FDR-corrected *p value* <0.05) [18]. Every ignorome gene was successfully assigned to one or more GO terms, including ion-channel activity, beta-amyloid binding, GABA metabolic process, neurotransmitter transport, neuron migration, synapse, and voltage-gated calcium channel complex (Table S5).

### Network properties of the ignorome

We defined large sets of genes that covary with members the ISE set (*n* = 306, see Methods) using several large brain transcriptome data sets (whole brain, cerebellum, hippocampus, nucleus accumbens, and prefrontal cortex) at thresholds of |*r*| = 0.75, 0.80, 0.85, 0.90, and 0.95. Remarkably, there is no correlation between the depth of literature coverage and the number of super-threshold covariates in any data set at any level. We conclude that connectivity of ISE genes in any of several well defined brain regions does not predict how well it has been studied. A few ignorome genes including *RUFY3*, *TRNP1*, *FAM81A*, *SLITRK4*, and *CNIH3* are among the top 20 most highly connected genes in the brain and have higher connectivity than extraordinarily well-studied genes such as *GABRB3*, *MBP*, *SYP*, and *SNCA*.

### Homolog properties of the ignorome

#### Paralog comparison

There is no significant correlation between paralog abundance and literature coverage for ISE genes (*r* = −0.08). The average numbers of known paralogs for 100 ignorome genes and 100 of the best-studied ISE genes are closely matched—2.2 and 3.0, respectively. Similar results were obtained for human orthologs. The correlation between paralog counts and literature coverage is very close to zero (−0.085).

#### Ortholog comparison

Using comparative data for 21 species in HomoloGene (ftp.ncbi.nih.gov/pub/HomoloGene)[19], there is a highly statistically significant positive correlation between ortholog number and literature coverage (*r* = 0.16) using the logarithm of the literature coverage to normalize the distribution. Ignorome members have slightly fewer orthologs with a slope of 0.4 orthologs per 10-fold increase in literature, but this difference while significant, only accounts for 2–3% of the variance. The average numbers of known orthologs for 100 ignorome genes and 100 of the best-studied ISE genes are—8.19 and 9.79, respectively.

#### Protein signature analysis

The correlation between the logarithm of the neuroscience literature and numbers of protein domains for all 650 ISE gene is again weakly positive (+0.7 domains per 10-fold increase in literature, *r^2^* = 4.2%) but is still highly significant (*p*<.0001). This trend is much more evident in the extremes of the distribution—the average domain count for the best studied set of 100 genes is more than twice that of the worst studied subset of 100 genes (*n* = 4.7 vs 2.0 domains). Twenty ignorome genes have no known protein signatures, whereas all well-studied ISE genes have at least one protein domain.

### Time series analysis of the ignorome

**Figure 4:**
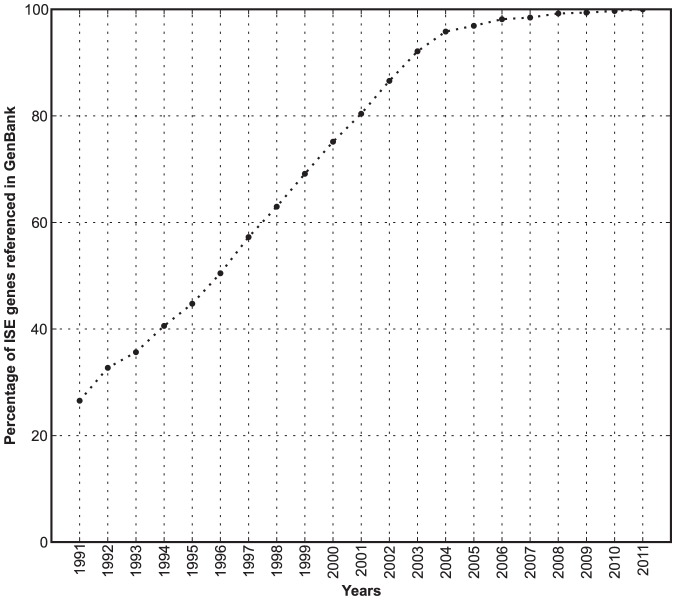
Year of discovery of genes. The x-axis represents the timeline. The line represents the perecentage of ISE genes whose mRNA or protein have been referenced into GenBank.

We evaluated whether sparse functional annotation and lack of scientific attention focused on the ignorome is simply a matter of discovery date and duration of study. For every ISE gene, we retrieved the year that the gene or its orthologs were first referenced in sequence databases including GenBank (ftp.ncbi.nlm.nih.gov/refseq/M_musculus/mRNA_Prot). In most cases, this date corresponds to the year the first cDNA clone was submitted to GenBank (ftp.ncbi.nlm.nih.gov/refseq/M_musculus/mRNA_Prot). As anticipated, the well-studied subset of genes was generally introduced earlier to the research community than the ignorome subset— with medians of 1988 and 2002, respectively (*p*<10^−18^, one sided *t* test). Forty percent of ignorome genes were introduced during the watershed sequencing years (2001–2003) for mouse and human genomes (Figure 4). However, it is serious concern that 60% of the ignorome have been well defined protein-coding genes for over a decade.

#### Rate of evaporation

**Figure 5:**
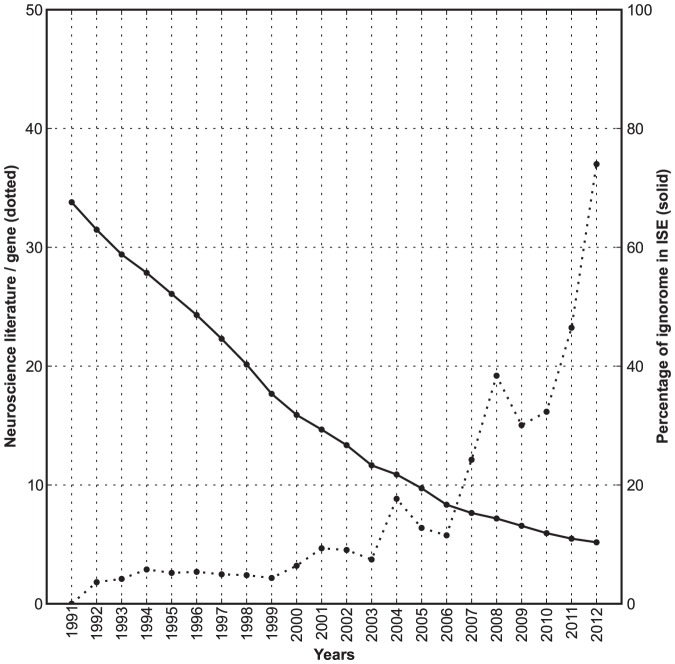
Shrinkage of the ignorome. The *x*-axis represents the timeline. The solid line represents the percentage of ignorome genes in the ISE brain set. The dotted line represents the number of neuroscience specific literature (in thousands).

Will untargeted and semi-random community research effectively remove the ignorome in the next few years? To address this question we calculated the rate at which the ignorome has shrunk over the past two decades? Our starting point for this analysis was 1991. At this early stage of genomics, two-thirds of our reference set of 648 ISE genes had no literature at all. This number has been reduced by 90% and only 67 genes are still part of an absolute ignorome with no neuroscience literature and almost no literature in an area of research. While the average rate of decrease was rapid between 1991 and 2000 (−25 genes/year), the rate has been lethargic over the past five years (−6.4 genes/yr, Figure 5). This trend is surprising given the sharp increase in the rate of addition to the neuroscience literature. As a result, the number of neuroscience articles associated with the elimination of a single ignorome gene has gone up by a factor of three between 1991 and 2012 (Figure 5). The rate at which the ignorome is shrinking is approaching an asymptote, and without focused effort to functionally annotate the ignorome, it will likely make up 40–50 functionally important genes for more than a decade.

### Genetic analysis of the ignorome

We used a reverse complex trait approach combined with a massive phenome data set in GeneNetwork [20] to evaluate the range of CNS and behavioral phenotypes that map downstream of ignorome genes. More specifically we exploited sequence variants in ignorome genes that are known to segregate in the BXD family of inbred strains [6], [21], [22]. We provide three concrete examples below, and we provide more specific instructions that can be used to apply this technique in the Methods section.

- Expression of *ELMOD1* is associated with a strong cis-eQTL in a massive RNA-seq brain data set that we generated (*UTHSC Mouse BXD Whole Brain RNA Sequence Exon Level RPKM*, see Methods). The LRS peaks at a value of 23 and the *D* allele is associated with high expression. *ELMOD1* is also a cis eQTL in several regional data sets, including the hippocampus (LRS = 43), hypothalamus (LRS = 21), prefrontal cortex (LRS = 37), striatum (LRS = 25), and cerebellum (LRS = 24). One likely causal variant is a ∼6200 bp long interspersed nuclear element that is present in all family members that inherit the *B* haplotype relative to the *D* wildtype haplotype. This analysis provides a reason to suspect that variants in *ELMOD1* may have downstream effects on higher order phenotypes. In support of this idea, four independent studies have shown that variation in locomotor activity maps to the *ELMOD1* locus on chromosome 9 (GeneNetwork traits: GN10566 [23], GN11518 [24], GN10132 [25], and GN10010 [26]). Hearing sensitivity also maps to this region (GN10654), and *ELMOD1* is a strong candidate gene for this trait given independent evidence that mutations in this gene cause hearing and balance deficits [27].
- Expression of *TMEM88B* is consistently associated with cis-eQTLs in whole brain (LRS = 12), hippocampus (LRS = 25), hypothalamus (LRS = 13), and striatum (LRS = 13). The *B* allele is associated with higher expression. A high impact missense SNP (*rs32361880*) in exon 1 substitutes a polar arginine (*D* haplotype) for a nonpolar glycine (*B* haplotype) within the transmembrane helix as predicted by TMHMM [28]. Several feeding and gustatory traits have been mapped to this part of chromosome 4, including GN10578 [29] and GN10475 [30]).These gustatory traits are already known to be caused by a mutant *D* allele of the *TAS1R3* sweet receptor [31]. However, handling-induced convulsion, GN10578 [29], is still a viable candidate trait that may be functionally linked to the missense mutation in *TMEM88B*.
- *DZANK1* is also consistently associated with cis-eQTLs in mouse brain (LRS = 17), hypothalamus (LRS = 16), amygdala (LRS = 32), and striatum (LRS = 23). *DZANK1* has a missense mutation that substitutes a valine (*D* haplotype) for an isoleucine (*B* haplotype). Traits that map to the *DZANK1* interval on chromosome 2 (139–150 Mb) include an assay for depression (GN11432, the tail suspension test [24]) and ambulatory activity (GN12890 [21]). Expression of *DZANK1* in amygdala covaries strongly with both of these traits (*r* = 0.70 and *r* = −0.77) and both are therefore downstream phenotypes that may well be associated with the *DZANK1* missense mutation and cis-eQTL.

## Discussion

After two decades of systematic genomic studies of the nervous system [35]–[37] nearly one-sixth (n∼106) of all genes with highly specific expression in the brain are still part of the core ignorome. The skewed distribution of literature may be due to the inherent differences in the importance of these genes, but we suspect that initial bias in discovery and social and scientific research momentum is a much more likely factor. Hoffman and Valencia [1] showed no correlation between a gene's impact in the scientific literature and it's centrality in protein-protein interactions, a finding that certainly is concordant with our main results. However, some skew in the literature could also be due to intrinsic differences among ISE genes, particularly the number of known protein domains. It is possible that the well-studied genes have still undiscovered characteristics that justify the extra affection that they continue to receive. Interestingly, we did not find any prominent differences between well studied and ignorome sets at any level of analysis, including expression selectivity, co-expression connectivity, and species conservation (paralog and ortholog abundance). We argue that that ignored genes will ultimately prove to be as important as the well-studied genes such as *MBP*, *GABRA1*, and *GRIN1*.

### Defining an ignorome

Ignoromes can be defined for whole systems or for single cell fractions. Ignoromes can also be calculated for different states such as a disease or response to a drug treatment. The only restriction in computing different types of ignoromes is that the literature pertaining to the relevant area of research must be sufficiently large. Large literature size will ensure a relatively smooth distribution of scores and adequate resolution and ranking of genes. For example, pancreatic beta cells (∼20,000 papers) and astrocytes (∼36,000 papers) have enormous literatures and ignoromes could be easily computed given the literature and numerous ‘omics’ data sets, even at the cellular level. In every case, careful manual curation will be essential to avoid errors introduced by ambiguous nomenclature or incorrect gene models. The methods and code that we provide in the Methods section should provide a start to systematically define what the blind spots in genome functional annotation.

Assuming that the literature distribution of ISE genes for any tissue follows a power law, the size of the ignorome will depend on number of genes that show intense and selective expression. For the expression data set that we used in our study [32], four other common tissues—testis, placenta, embryonic stem cells, and heart—also have comparatively large numbers of ISE genes. In contrast, tissues with modest cellular diversity or widely shared functions will often have smaller numbers of ISE genes and may also have smaller ignoromes. However, since the ignorome is also a function of literature coverage, there will be important exceptions and tissues with small numbers of ISE genes may have large ignoromes.

Depending on the goal of a study or system of interest, different quantitative metrics can be used to evaluate the ‘genetic’ or biological importance of genes. Expression selectivity, sequence conservation or centrality in protein networks are some characteristics that can be used to rank potential biological importance. Whole transcriptome data sets have advantages. The first of these is comprehensiveness—the availability of expression data for nearly all protein-coding genes. In contrast, mammalian protein interactomes are still incomplete and many proteins lack interaction data. The second, almost paradoxical advantage of expression data is the lack of association between a gene's expression selectivity score and its literature coverage. Expression selectivity scores are computed without reference to the literature. In contrast, specific protein–protein interactions are frequently reported in the literature. As a result, proteins with high numbers of interacting partners will obviously have high literature counts. For expression data, the literature is only minimally “contaminated” by large ‘omics’ studies and literature contaminants are easily removed.

Using tissue-selectivity scores as a metric overlooks genes that have widespread expression but that have highly specific function. For example, *BDNF* is expressed in many tissues [33], [34], including the brain, prostate, kidney and eyes, but is known to play an important role in development and adult brain function.

In this study we have defined a core CNS ignorome using stringent criteria, and the ignorme genes in Table S5 have at most a single article link in PubMed. Broadening inclusion criteria to include genes with as many as five articles could be easily justified and would expand the brain ignorome to ∼200 genes—almost one-third of the ISE brain set. In this study we have also applied a very high expression selectively criterion—8-fold higher expression in brain than in other tissue. This criterion could easily be relaxed to 4-fold, and the size of the brain ignorome would double. As shown in Figure 1, half of all brain ISE genes absorb only 1% of the neuroscience literature and from this vantage point could easily be defined as below the poverty line. Appropriate thresholds to define an ignorome will differ greatly depending on research context. The threshold we have used is suitable for highlighting only the most blatantly neglected starving genes that deserve much more research attention.

### Absolute versus relative ignoromes

It is practical to calculate global and local ignoromes for a given system. The global ignorome consists of genes with no literature coverage whatsoever. In contrast a local ignorome consists of genes that may have some literature coverage but no or very little literature coverage with respect to a particular system. In this paper, we focused on the brain ignorome but calculated both global and local (neuroscience-specific) ignoromes. We found that only a few of the local brain ignorome genes, including *COPG, SMAP1, JPH4* and *MAGEE1*, have been studied to some extent outside of neuroscience. Thus our stringent brain ignorome is nearly equivalent to a global ignorome.

### Annotating the ignorome

We have used diverse resources to add useful functional annotation to several core ignorome genes. We exploited InterPro and Pfam, *in situ* hybridization expression data from the Allen Brain Institute, and GeneNetwork's massive compendium of phenotype and transcript expression data. This overlay of bioinformatic and genetic annotation, summarized in Table S5, is helpful as an initial pointer, but even when we combine these resources we cannot provide mechanistically validated functional annotation. For example, information on protein domains is helpful as a clue to function, but domains such as the leucine-rich repeats have been identified in a large number of functionally unrelated proteins [33]. As made clear by our retrospective analysis of the brain ignorome going back twenty years (Figure 5), molecular and genetic studies are essential to confidently remove a gene from the list.

### Network properties of the ignorome versus well-studied genes

Ignorome genes and well-studied genes showed no connectivity difference in brain co-expression networks. This suggests that ignorome genes may well prove to be as important as well-studied genes. Literature coverage of ISE genes showed a very weak but statistically significant correlation with both ortholog and protein domain numbers. But none of these factors accounted for more than 5% of the variance observed in literature coverage.

### Potentially important brain ignorome members

We conclude by highlighting a few examples of potentially important brain ignorome genes that by definition have minimal literature, but that already have intriguing, if tentative, links to neurological and psychiatric disorders.

- *CSMD3* encodes a transmembrane protein selectively expressed in adult and fetal brains and is a strong candidate for autism spectrum disorders [35] and benign adult familial myoclonic epilepsy [36]. Expression is extremely intense in the dorsomedial hypothalamic nucleus and hippocampus but weak in cerebellum. No clear function has been assigned to this gene, but it mRNA covaries well (*r* = 0.72) in mouse hippocampus with a voltage-gated sodium channel (*SCN3A*) that itself is linked to autism and epilepsy [37]. The expression of *CSMD3* in human forebrain [38] is strongly linked with genes involved in GABAergic interneuron fate commitment (adjusted *p* (adjP) = 0.0039) and neuron development (adjP = 0.010).
- *CSRNP3* is expressed almost exclusively in brain and pineal gland. *CSRNP3* has cell death-inducing activities and is predicted to play role in brain development [39]. *Csrnp3* mRNA covaries well in mouse hippocampus with ataxin 1(*ATXN1, r* = 0.65) and *PTEN* (*r* = 0.64). *ATNX1* is linked with spinocerebellar ataxia 1[40] and *PTEN* is linked with glioblastoma, autism, and several other syndromes[41], [42]. In human forebrain, *CSRNP3* covaries well with genes involved in alcohol transmembrane transporter activity (adjP = 0.018).
- *TCBA1* is well conserved among mammalian species. The human ortholog is expressed exclusively in brain and thymus [43]. SNPs in and around *TCBA1* are linked to neurological phenotypes [44] and are associated with developmental delay [43]. *TCBA1* covaries remarkably well with *APP* in forebrain of mouse and human (*r* = 0.75 and *r* = 0.65).
- Nine genes from TAFA family, including *FAM123A, FAM123C, FAM131B, FAM155A, FAM171B, FAM189A1, FAM19A1, FAM19A2,* and *FAM81A*. All nine are highly expressed in specific regions of the brain and they have been postulated to function as neurokines [13].
- *FRRS1L* is the human ortholog of the murine *6430704M03Rik* gene. It is a novel brain-specific transcript that has been linked to familial dysautonomia [45]. It covaries well in mouse hippocampus with *GRIA1* (*r* = 0.4) and *SNCA* (*r* = −0.43). *GRIA1* is linked with spatial learning and memory [46] whereas *SNCA* is linked with Parkinson disease [47]. In human forebrain, the expression of this gene is strongly linked with neurotransmitter secretion (adjP  = .03).
- *DIRAS1* and *DIRAS2* belong to DIRAS gene family. *DIRAS1* is likely to be a neural tumor suppressor [48], and *DIRAS2* is associated with an adult form of attention deficit hyperactivity disorder [49]. *DIRAS1* in human forebrain is strongly linked with genes involved in nervous system development (adjP  = 0.008) and neurofilament cytoskeleton organization (adjP  = 0.004). *DIRAS2* is strongly linked with genes involved in hydrogen ion transmembrane transporter activity (adjP  = 0.043).

## Materials and Methods

### Generating expression data sets for different tissues

In order to estimate the relative selectivity of expression of genes in the brain versus other tissues, we dissected 26 organs and tissue types from young animals belonging to two fully inbred strains of mice —C57BL/6J and DBA/2J. (All data are available at the following link: http://genenetwork.org/dbdoc/ILM_B_D_CNS_GI_avg_0508.html.) Approximately 100 mg of each tissue was homogenized in 1 ml of RNA STAT-60 reagent. The purity of RNA was determined and in all cases the 260/280 ratio was between 1.9 and 2.1. Samples were amplified with the Illumina TotalPrep RNA kit and run on MouseWG-6 v2.0 arrays. Data were normalized using the rank invariant method. The variance across arrays was stabilized by converting values to z scores. These z scores have some disadvantages: half of all values are negative, and the range of values does not correspond to the original log2 values. To avoid these two problems we doubled z scores and added an offset of 8 units. The mean of these modified z scores is therefore 8 and the standard deviation is 2. Values are reasonably close to the original log2 scale and a two-fold difference in mRNA concentration corresponds roughly to a 1 unit [50]. Expression levels below 6 are close to background noise levels. We also exploited two other multiple organ expression data sets. The first is an Affymetrix data downloaded from NCBI Gene Expression Omnibus (www.ncbi.nlm.nih.gov/geo/query/acc.cgi?acc=GSE9954) [32] consists of transcriptome estimates generated across 19 organs and tissues from young adult C57BL/6 (3 to 5 males and females), whole bodies of E16 mice, placenta, and embryonic stem cells derived from C57BL/6JOla. We preprocessed the data using robust multichip analysis (RMA). We standardized expression values as described above for Illumina data. The second data set is part of the BioGPS suite (www.biogps.org) [51], [52]. BioGPS was used to independently confirm the selectivity of ISE gene expression in brain. All animal work was conducted according to an approved animal use protocol and in accordance with procedures approved by the Institutional Animal Care and Use Committee at UTHSC.

### Intensely and selectively expressed genes in brain

We computed a brain selectivity score for every probe by dividing its expression in brain by its average expression across all other tissues. Probes (genes) were operationally defined as preferentially expressed in brain if this ratio was greater than 8. Many probes(genes) with high expression in brain also have high expression in testis and retina [53]. As a result, we relaxed our criteria for inclusion to allow for a limited level of expression in no more than 5 of 22 comparisons. The final list used for computing a generic brain ignorome is therefore selective rather than exclusive.

When two or more probe identifiers mapped to the same gene identifiers, we retained one with the highest brain selectivity. We excluded probe sets that (1) mapped to multiple locations (2) belong to a non-protein-coding gene. We also removed probe sets containing SNPs and small indels between B6 and D2 to avoid false expression estimates caused by hybridization artifacts [54].

We converted brain selectivity scores into relative brain selectivity scores by dividing them with the highest brain selectivity score for that array. The resulting relative scores for both arrays ranged between 0 and 1 and were merged together to form a single data set. In case where the gene was common to both arrays, the highest relative selectivity score was retained.

### Expression analysis using RNA-seq

Total RNA was isolated from the whole brains of adult mice using RNA STAT-60 (Tel-Test Inc) as described in Li, Mulligan, and colleague [6]. RNA samples with RNA integrity number greater than 8 were used to generate transcriptome libraries, which were sequenced on the SOLiD platform. Short sequence reads were analyzed using Applied Biosystems whole transcriptome software tools. Reads were mapped to the C57BL/6J reference genome (NCBI build 37) with a minimum mapping quality of 25. We were aware of the allelic bias problem in aligning reads of DBA2/J (D2) origin to the B6 reference genome. However, our purpose of using RNA-seq results was to determine exact 3′ UTR length rather than quantitative expression difference between B6 and D2 transcriptome. Expression estimates were calculated as the reads per kilobase of transcript per million mapped reads (RPKM). This metric normalizes a transcript's read count by both its length and the total number of mapped reads in the sample.

### Validation of annotation of ISE brain genes

Annotations of microarray probes are still surprisingly poor. Most of the probes we have exploited target the 3′ UTRs of mRNAs, and unfortunately, annotations of 3′ UTRs of genes with high expression in the brain are especially poor due to their extreme length [55]. We generated deep RNA-seq data (>1 billion 50 nt oriented tags from rRNA-minus whole brain RNA) to reliably determine the maximal length of 3′ UTRs of mRNAs in the brain and the full extent of the cognate genes. All of our summed brain RNAseq tag count data are accessible at ucscbrowser.genenetwork.org and whole brain gene expression level expression data (RPKM) for 28 strains of mice are accessible at www.genenetwork.org. Go to www.genenetwork.org. Select “Mouse” as species, “BXD” as group, “Brain mRNA” as type, and “UTHSC Mouse BXD Whole Brain RNA Sequence Exon Level (Nov12) RPKM” as dataset. This RNA-seq data enabled us to accurately confirm and revise gene models and correct annotation errors, particularly those related to long brain specific 3′ UTRs.

### Collecting gene and homolog information for ISE genes

We used annotation files from Affymetrix and Illumina to map probes to genes. We also manually curated probes using RNA-seq data. We used mouse-human and mouse-rat ortholog files from Mouse Genome Informatics (www.informatics.jax.org) and the HomoloGene file from NCBI (ftp.ncbi.nih.gov/pub/HomoloGene/current) to get corresponding gene identifiers for mouse, human and rat. HomoloGene was also used to compute the number of orthologs for ISE genes.

### Constructing gene nomenclature table and selecting ambiguous gene names

We retrieved official gene names, symbols, aliases, and other designation, including protein names for genes in mouse and their orthologs from NCBI (ftp.ncbi.nlm.nih.gov/gene/DATA/GENE_INFO/Mammalia). A total of 7,563 gene aliases and other designations are available for 648 ISE genes. We selected the subset of ambiguous gene names as follows. We downloaded a list of English words (www.mieliestronk.com/wordlist.html) and used this list to select gene names associated with common English language words—for example *CALM*, *Brain-1*. We also selected gene names that were too short, for example, *‘sp’, ‘F3’, ‘A-2’*. We used biomedical acronym resolver ARGH (http://etblast.vbi.vt.edu/argh/index.shtml) and selected gene names that are also acronyms for other non-relevant biomedical terms. We found 974 ambiguous gene names (symbols or aliases), and of these 118 gene names match English words; 155 gene names have been used with multiple senses in PubMed; 701 gene names that are too short for non-ambiguous retrieval.

### Generating gene-specific informative keywords

We collected official full gene and protein names for genes in mouse, human, and rat as described above. We split compound gene and protein names into constituents. Uninformative words such as “protein”, “factor”, “family”, and “binding” were removed. We also removed words that occurred at high frequency (>20 occurrences) from the collection. For every gene we manually selected one of its constituent words that occur with low frequency.

### Generating neuroscience specific keywords

We extracted MeSH terms from ∼50,000 articles belonging to 40 different neuroscience journals. For this purpose we used the title abbreviation tag (TA) to limit PubMed searches to neuroscience journals and counted frequencies of MeSH terms. The same was done for ∼150,000 non-neuroscience articles. We selected MeSH terms that occurred at a relatively higher frequency (>20%) in neuroscience articles and relatively low frequency in (<1%) non-neuroscience articles. The final list of 155 terms used to define the neuroscience literature (Appendix S2) consists of 129 computationally extracted terms (e.g., postsynaptic, prefrontal, hippocamp*, amygdala, psychomotor, somatosensory, neuropsychological, gyrus, metabatropic, parkison*, bicuculline) and 26 relatively generic neuroscience terms (e.g., neuron, axon, brain, autis* CNS, nerve, glial, seizure).

### Generating a list of noisy articles

We extract the subset of genes that are associated with more than 50 genes in PubMed using *gene2pubmed* (ftp.ncbi.nlm.nih.gov/gene/DATA/gene2pubmed.gz). While some of these 800 papers have useful functional annotation for ignorome genes, a manual overview of ∼50 of these articles shows that they add minimal if any significant insight into brain function or disease.

### Estimating literature coverage

In order to estimate literature coverage for each gene we developed a concatenated PubMed query using NCBI EUtils. The query had the following structure:

1. *Subject of the query*: All unambiguous aliases, gene symbols, and gene names were joined using the OR operator to create a comprehensive query. In those cases in which gene names were ambiguous, they were joined with gene-specific keywords.
2. *Neuroscience membership*: The set of 155 neuroscience-specific keywords were combined with the query above.
3. *Range of species*: The search term “AND (*Mus musculus* OR *Homo sapiens* OR *Rattus norvegicus*)” was added to further restrict the search.
4. *Range of text fields*: The title and abstract tag (TIAB) was used to avoid inclusion of irrelevant fields.

We provide one full example of this complex concatenated query for the gene synapsin 1 (*Syn1*) in Appendix S1.

### Calculating an ignorome score

For every ISE gene we calculated two fractional scores. First was the fraction of ISE genes with higher relative brain selectivity than the gene and second was the fraction of ISE genes with less neuroscience literature size. Finally, we calculated an ignorome score by taking the harmonic mean of the above two fractions.

### Estimating paralog abundance

We downloaded *M. musculus* and *H. sapiens* paralog data from Ensembl BioMart (www.ensembl.org/info/data/biomart.html) [56], [57]. We only used paralogs defined by Ensembl as—*within_species_paralogs* to compute numbers of paralogs for each gene.

### Genetic correlation analysis of ignorome genes

We used a BXD whole-brain Affymetrix M430 2.0 data set consisting of ∼44,000 probe sets. To avoid spurious correlations with poorly expressed transcripts, we removed all probe sets with expression <8 units. We also removed probe sets containing SNPs and small indels between B6 and D2 to avoid false correlation caused by hybridization artifacts [54]. We computed Pearson product-moment correlations between the ignorome genes (probe sets) and other probe sets in the brain (∼37,000) using a data set consisting of at least 30 different strains of mice (see http://genenetwork.org/webqtl/main.py?FormID=sharinginfo&GN_AccessionId=123) [18]. To exclude spurious high correlations caused by linkage, we eliminated data for pairs of transcripts that are chromosomal neighbors (within 5 Mb of each other).

### Reverse complex trait analysis

We selected a subset of 15 ignorome genes that are *cis*-modulated (*cis-*eQTL) in one or more of 35 brain expression data sets generated for the BXD family [54]. The following criteria were used: (1) an LRS score >12, (2) expression >7, and (3) moderate or high impact coding variants. For reverse complex trait analysis we exploited GeneNetwork.org, a database consisting of ∼3,800 phenotypes known to vary widely across the BXD family. Phenotypes that mapped to within 5 Mb of each ignorome gene with an LRS >10 were defined as candidate traits using the following search string: “LRS =  (10 999 ChrZ XX YY)” where Z is replaced with the correct chromosome and XX and YY are genome positions (in megabases) located 5 Mb proximal and distal to the ignorome gene.

## Supporting Information

Figure S1 **Location of 1443205_at probe set.** Snapshot of the brain RNAseq data confirming the location of the probe 1443205_at in the distal 3′ UTR of the *Gabrb1* gene. BR+BXD All track represents the expression on the plus strand.
(XLS)

Table S1 (XLS)

Table S2 (XLS)

Table S3 (XLS)

Table S4 (XLS)

Table S5 (XLS)

Appendix S1 (DOCX)

Appendix S2 (DOCX)
