# Supplementary figures and images for: Functionally Enigmatic Genes: A Case Study of the Brain Ignorome
Source: PLoS One. 2014 Feb 11;9(2):e88889. doi: 10.1371/journal.pone.0088889 (PMC3921226; doi:10.1371/journal.pone.0088889)

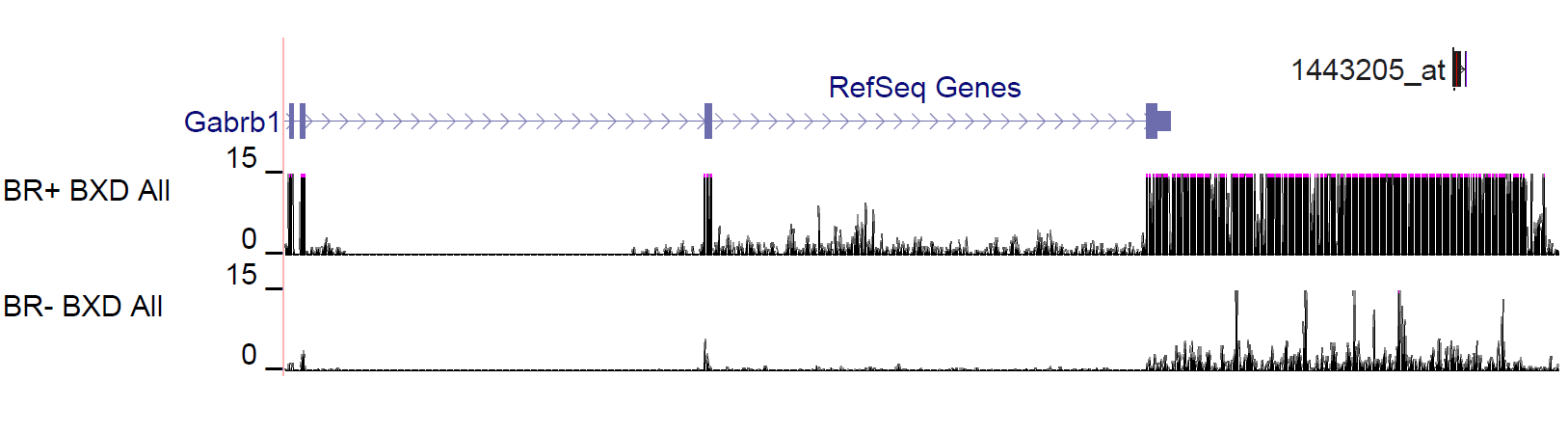

Supplement: Figure S1 — Location of 1443205_at probe set. Snapshot of the brain RNAseq data confirming the location of the probe 1443205_at in the distal 3′ UTR of the Gabrb1 gene. BR+BXD All track represents the expression on the plus strand. (XLS) [file pone.0088889.s001.xls]
