# Supplementary material for: Functionally Enigmatic Genes: A Case Study of the Brain Ignorome
Source: PLoS One. 2014 Feb 11;9(2):e88889. doi: 10.1371/journal.pone.0088889 (PMC3921226; doi:10.1371/journal.pone.0088889)
Supplement: Appendix S1 — (DOCX) [file pone.0088889.s007.docx]

**Appendix S1**

We provide one full example of this complex concatenated query for the gene synapsin 1 (*Syn1*). The query below will generate 965 PubMed identifiers, all of which are in neuroscience.

http://eutils.ncbi.nlm.nih.gov/entrez/eutils/esearch.fcgi?db=pubmed&term=("syn1"[TIAB] OR "synapsin+i"[TIAB] OR "syn+1"[TIAB] OR "synapsin+1"[TIAB] OR "syn1a"[TIAB] OR "syn1b"[TIAB] OR "syni"[TIAB] OR "brain+protein+4+1"[TIAB] OR "synapsin+1"[TIAB])+ AND+(accumbens[TIAB] OR afferents[TIAB] OR amacrine[TIAB] OR amygdala[TIAB] OR amyotrophic[TIAB] OR antiparkinson[TIAB] OR antipsychotic[TIAB] OR aplysia[TIAB] OR apomorphine[TIAB] OR astrocyt*[TIAB] OR alzheimer*[TIAB] OR autis*[TIAB] OR axonal[TIAB] OR axon*[TIAB] OR brain[TIAB] OR brains[TIAB] OR behavioral[TIAB] OR benzodiazepines[TIAB] OR bicuculline[TIAB] OR brainstem[TIAB] OR callosum[TIAB] OR cannabinoids[TIAB] OR carbocyanines[TIAB] OR catesbeiana[TIAB] OR caudate[TIAB] OR cerebellar[TIAB] OR cerebr*[TIAB] OR cerebellum[TIAB] OR chemoreceptor[TIAB] OR cingul*[TIAB] OR cortex[TIAB] OR collicul*[TIAB] OR convulsants[TIAB] OR CNS[TIAB] OR decerebrate[TIAB] OR demyelinating[TIAB] OR denervation[TIAB] OR dentate[TIAB] OR dendrit*[TIAB] OR dextroamphetamine[TIAB] OR diencephalon[TIAB] OR dopaminergic[TIAB] OR dynorphins[TIAB] OR dyskinesia[TIAB] OR dystonia[TIAB] OR endocannabinoids[TIAB] OR enkephalins[TIAB] OR entorhinal[TIAB] OR epileps*[TIAB] OR epilepticus[TIAB] OR fluoxetine[TIAB] OR forelimb[TIAB] OR gabaa[TIAB] OR gabaergic[TIAB] OR gerbillinae[TIAB] OR glial[TIAB] OR gliosis[TIAB] OR glutamatergic[TIAB] OR gyrus[TIAB] OR haloperidol[TIAB] OR hippocamp*[TIAB] OR huntington[TIAB] OR hydroxydopamines[TIAB] OR hyperalgesia[TIAB] OR hypothalam*[TIAB] OR interneurons[TIAB] OR intracranial[TIAB] OR iontophoresis[TIAB] OR laterality[TIAB] OR limbic[TIAB] OR locomotor[TIAB] OR medul*[TIAB] OR mesencephal*[TIAB] OR metabotropic[TIAB] OR methamphetamine[TIAB] OR microglia[TIAB] OR midbrain[TIAB] OR monoamines[TIAB] OR motoneurons[TIAB] OR myelinated[TIAB] OR myelin*[TIAB] OR nervous[TIAB] OR nerve[TIAB] OR neocortex[TIAB] OR neocortical[TIAB] OR neostriatum[TIAB] OR neurite*[TIAB] OR neuro*[TIAB] OR neuron*[TIAB] OR neural[TIAB] OR neurodegener*[TIAB] OR neurofi*[TIAB] OR neurogen*[TIAB] OR neurokinin[TIAB] OR neuropathy[TIAB] OR neurophysi*[TIAB] OR neuropil[TIAB] OR neuroprotect*[TIAB] OR neuropsychological[TIAB] OR neurotensin[TIAB] OR neurotoxi*[TIAB] OR neurotrophin[TIAB] OR neuromuscular[TIAB] OR nigra[TIAB] OR nocicept*[TIAB] OR noradrenergic[TIAB] OR oblongata[TIAB] OR oculomotor[TIAB] OR odor*[TIAB] OR oligodendro*[TIAB] OR orbitofrontal[TIAB] OR oxidopamine[TIAB] OR pituitary*[TIAB] OR pallidu*[TIAB] OR parahippocamp*[TIAB] OR paraventricular[TIAB] OR parkinson*[TIAB] OR pellucidum[TIAB] OR perforant[TIAB] OR postsynaptic[TIAB] OR prefrontal[TIAB] OR preganglionic[TIAB] OR premotor[TIAB] OR preoptic[TIAB] OR presynaptic[TIAB] OR psychomotor[TIAB] OR psychophysics[TIAB] OR psychophysiologic[TIAB] OR purkinje[TIAB] OR rhodopsin[TIAB] OR rhombencephalon[TIAB] OR schwann[TIAB] OR sciatic[TIAB] OR seizure*[TIAB] OR sensorimotor[TIAB] OR serotonergic[TIAB] OR somatosensory[TIAB] OR spatiotemporal[TIAB] OR spinocerebellar[TIAB] OR stereotaxic[TIAB] OR striat*[TIAB] OR subcortical[TIAB] OR substantia[TIAB] OR subthalamic[TIAB] OR synaps*[TIAB] OR synaptophysin[TIAB] OR synaptosom*[TIAB] OR tachykinins[TIAB] OR tegmental[TIAB] OR tegmentum[TIAB] OR telencephalon[TIAB] OR thalamus*[TIAB] OR thalamic[TIAB] OR unmyelinated[TIAB] OR vocalization[TIAB])+ AND+(Mus+Musculus OR Homo+Sapiens OR Rattus+Norvegicus)&retmax=10000
