# Supplementary material for: Functionally Enigmatic Genes: A Case Study of the Brain Ignorome
Source: PLoS One. 2014 Feb 11;9(2):e88889. doi: 10.1371/journal.pone.0088889 (PMC3921226; doi:10.1371/journal.pone.0088889)
Supplement: Appendix S2 — (DOCX) [file pone.0088889.s008.docx]

**Appendix S2**

List of neuroscience specific keywords

| accumbens |
| --- |
| afferents |
| amacrine |
| amygdala |
| amyotrophic |
| antiparkinson |
| antipsychotic |
| aplysia |
| apomorphine |
| astrocyt* |
| alzheimer* |
| autis* |
| axonal |
| axon* |
| brain |
| brains |
| behavioral |
| benzodiazepines |
| bicuculline |
| brainstem |
| callosum |
| cannabinoids |
| carbocyanines |
| catesbeiana |
| caudate |
| cerebellar |
| cerebr* |
| cerebellum |
| chemoreceptor |
| cingul* |
| cortex |
| collicul* |
| convulsants |
| CNS |
| decerebrate |
| demyelinating |
| denervation |
| dentate |
| dendrit* |
| dextroamphetamine |
| diencephalon |
| dopaminergic |
| dynorphins |
| dyskinesia |
| dystonia |
| endocannabinoids |
| enkephalins |
| entorhinal |
| epileps* |
| epilepticus |
| fluoxetine |
| forelimb |
| gabaa |
| gabaergic |
| gerbillinae |
| glial |
| gliosis |
| glutamatergic |
| gyrus |
| haloperidol |
| hippocamp* |
| huntington |
| hydroxydopamines |
| hyperalgesia |
| hypothalam* |
| interneurons |
| intracranial |
| iontophoresis |
| laterality |
| limbic |
| locomotor |
| medul* |
| mesencephal* |
| metabotropic |
| methamphetamine |
| microglia |
| midbrain |
| monoamines |
| motoneurons |
| myelinated |
| myelin* |
| nervous |
| nerve |
| neocortex |
| neocortical |
| neostriatum |
| neurite* |
| neuro* |
| neuron* |
| neural |
| neurodegener* |
| neurofi* |
| neurogen* |
| neurokinin |
| neuropathy |
| neurophysi* |
| neuropil |
| neuroprotect* |
| neuropsychological |
| neurotensin |
| neurotoxi* |
| neurotrophin |
| neuromuscular |
| nigra |
| nocicept* |
| noradrenergic |
| oblongata |
| oculomotor |
| odor* |
| oligodendro* |
| orbitofrontal |
| oxidopamine |
| pituitary* |
| pallidu* |
| parahippocamp* |
| paraventricular |
| parkinson* |
| pellucidum |
| perforant |
| postsynaptic |
| prefrontal |
| preganglionic |
| premotor |
| preoptic |
| presynaptic |
| psychomotor |
| psychophysics |
| psychophysiologic |
| purkinje |
| rhodopsin |
| rhombencephalon |
| schwann |
| sciatic |
| seizure* |
| sensorimotor |
| serotonergic |
| somatosensory |
| spatiotemporal |
| spinocerebellar |
| stereotaxic |
| striat* |
| subcortical |
| substantia |
| subthalamic |
| synaps* |
| synaptophysin |
| synaptosom* |
| tachykinins |
| tegmental |
| tegmentum |
| telencephalon |
| thalamus* |
| thalamic |
| unmyelinated |
| vocalization |
